# Polyamine Metabolism, Photorespiration, and Excitation Energy Allocation in Photosystem II Are Potentially Regulatory Hubs in Poplar Adaptation to Soil Nitrogen Availability

**DOI:** 10.3389/fpls.2020.01271

**Published:** 2020-08-26

**Authors:** Yanbo Hu, Manzer H. Siddiqui, Chunming Li, Luping Jiang, Heng Zhang, Xiyang Zhao

**Affiliations:** ^1^ State Key Laboratory of Tree Genetics and Breeding, School of Forestry, Northeast Forestry University, Harbin, China; ^2^ Forestry College, Beihua University, Jilin, China; ^3^ Department of Botany and Microbiology, College of Science, King Saud University, Riyadh, Saudi Arabia; ^4^ Institute of Forestry Science, Heilongjiang Academy of Forestry, Harbin, China

**Keywords:** exponential fertilization, nitrogen utilization efficiency, photorespiration, polyamine, polyamine acetylation, *Populus*

## Abstract

Nitrogen fertilization is common for poplar trees to improve growth and productivity. The utilization of N by poplar largely depends on fertilizer application patterns; however, the underlying regulatory hubs are not fully understood. In this study, N utilization and potentially physiological regulations of two poplar clones (XQH and BC5) were assessed through two related experiments (i: five levels of N supply and ii: conventional and exponential N additions). Poplar growth (leaf area) and N utilization significantly increased under fertilized compared to unfertilized conditions, whereas photosynthetic N utilization efficiency significantly decreased under low N supplies. Growth characteristics were better in the XQH than in the BC5 clone under the same N supplies, indicating higher N utilization efficiency. Leaf absorbed light energy, and thermal dissipation fraction was significantly different for XQH clone between conventional and exponential N additions. Leaf concentrations of putrescine (Put) and acetylated Put were significantly higher in exponential than in conventional N addition. Photorespiration significantly increased in leaves of XQH clone under exponential compared to conventional N addition. Our results indicate that an interaction of the clone and N supply pattern significantly occurs in poplar growth; leaf expansion and the storage N allocations are the central hubs in the regulation of poplar N utilization.

## Introduction

Soil nitrogen (N) availability is one of primary factors determining plant N status. Soil N, which exists in multi forms (such as nitrate, ammonium, and amino acids), is highly mobile. Soil N availability is variable in natural field conditions due to biotic (*e.g.*, microbial consumption) and abiotic (*e.g.*, rainwater leaching) factors (Kant et al., 2011). Under the background of global warming, N limitation on plant growth has been predicted to be a long-term challenge for dryland vegetation systems (Hooper and Johnson, 1999). N uptake by roots is significantly restricted under severe drought stress even if soil N is sufficient (Hu and Chen, 2020). Moreover, N limitation is common in plants grown in infertile soil or good soil with short-rotation. Limitations of soil N availability result in inhibition of lateral root initiation, leaf development (*e.g.*, leaf number and leaf area) even whole plant growth (Yeh et al., 2000). Deficiency of N in plant is commonly accompanied with significant alternations of gene expressions, metabolic and physiological processes (Lea et al., 2007; Hu et al., 2014; Song et al., 2019a; Song et al., 2019b; Hu et al., 2020). How to improve plant adaptation to N deficiency is a high concern in dryland agriculture.

Conventional N fertilization, which delivers fertilizers at a constant rate during the growth period (Hu et al., 2015), is preferred by farmers to improve plant N use efficiency and overall productivity. In the past decades, excess N fertilizer was applied into global agricultural systems (Good and Beatty, 2011). However, crops can utilize only 30–40% of the supplemental N (Raun and Johnson, 1999). High N supply can produce inhibitory effects on root growth (*e.g.*, lateral root elongation) (Zhang et al., 2007), and reduce plant N utilization efficiency (Coleman et al., 2006). Appropriate fertilization managements are very crucial for crops to obtain maximum N utilization efficiency. Most of the plants obey the S-shaped growth pattern (*i.e.*, lag phase, log phase, and stationary phase). Correspondingly, plant N demand is relatively less at an initial growth stage (lag phase), and rapidly increases in the log phase, and slightly decreases in the stationary phase. Exponential N fertilization has been established on a basis of plant “S” growth pattern, so it was considered an optimal fertilization model for the plants such as *Pinus monticola* (Dumroese et al., 2005) and trembling aspen (Pokharel and Chang, 2016). However inconsistent results were reported in some woody species such as *Eucalyptus globulus* and hybrid poplar; for example, at a lower N-fertilization rate, plant morphological and nutritional traits were similar between exponentially and conventionally fertilized seedlings (CloseI et al., 2005; Zabek and Prescott, 2007). Plant responses to N addition are highly plastic due to the genetic-dependent (such as species preference to N form) and -independent (such as N addition patterns) factors (da Silveira Pontes et al., 2010). The applicability and efficacy of exponential models for different woody species are still largely unknown.

Leaves are the major N sink of plants supplied with N fertilizers. Leaf N is primarily located in the chloroplasts (in form of chloroplast proteins). During chloroplast development, polyamines (PAs) serve as an N source for the synthesis of chlorophyll and chloroplast proteins (Sobieszczuk-Nowicka and Legocka, 2014). Polyamines are small, polycationic molecules mainly present in chloroplasts and photosynthetic subcomplexes including thylakoid membranes, photosystem II complex and light-harvesting complex (Kotzabasis et al., 1993). Considerable evidence emphasized the importance of PAs in modulating the functionality and efficiency of photosynthetic apparatus (Shu et al., 2012; Sobieszczuk-Nowicka and Legocka, 2014). PAs can stimulate leaf conversion of protochlorophyllide to chlorophyllide (Beigbeder et al., 1995) and positively modulate photosystem II activity and linear electron transfer (Ioannidis and Kotzabasis, 2007). Exogenous PAs have positive impacts on leaf photochemical processes in plant response to abiotic stresses (Shu et al., 2012). Moreover, PAs participate in the modulation of energy production and dissipation processes of the chloroplast (Ioannidis et al., 2012; Kotakis et al., 2014). Small changes of plastid-associated PAs can fine tune proton motive force and ATP production in the thylakoids (Ioannidis and Kotzabasis, 2014). In higher plants, free PAs are the main forms present in plant organs (Chen et al., 2019). Free PAs are implicated in the regulation of C and N metabolisms (such as amino acids and tricarboxylic acid cycle). Therefore, PAs are significantly important in leaf physiological responses to N supply. However, to date, the information of PAs-associated plant response to exponential N addition is unavailable.

Photorespiration is physiologically important in the regulation of photosynthetic carbon (C) and N metabolism in leaves (Wang et al., 2014). In early studies (*e.g.*, Zelitch, 1973), photorespiration is considered an excess energy dissipating process, which protects the photosynthetic apparatus from stress damages. So lowering photorespiration was operated as a potential pathway to increase leaf C fixation and plant productivity. However, increasing evidence suggests the importance of photorespiration in the regulation of N metabolism (*e.g.*, having a positive correlation with nitrate assimilation, Bloom, 2015; stimulating the fixation of C into amino acids, Busch et al., 2018). Photorespiration was implicated in plant adaptation to low N stress (Tang et al., 2018). Under NO_3_^−^ supply (with one case of sunflower plants, Busch et al., 2018), photorespiration significantly increased, being accompanied with improvements in leaf N assimilation and CO_2_ uptake. Moreover, photorespiratory pathway significantly varies with leaf age (Gjerstad, 1975). For example, the photorespiration rate is high in developing poplar leaves, whereas the value declines in the matured leaves. Leaf maturation and senescence are closely correlated with N availability (Hallik et al., 2009). Photorespiratory pathway is a potentially regulatory hub in plant adaptation to N availability.

Poplar trees are short-rotation woody plants in the northern hemisphere (Hart et al., 2015). Poplar plantations have high biomass production at the cost of high water and N consumption (Rockwood et al., 2004). Soil N (particularly NO_3_^−^) losses have been reported in poplar plantations (*e.g.*, *P. maximowiczii* × *P. nigra*, Balasus et al., 2012; *Populus tremula* L. × *P. tremuloides* Michx., Rytter and Rytter, 2018) under the short-rotation regimes. The limitation of soil N availability is an important factor influencing the sustainability of high biomass production of the short-rotation poplar plantations. Appropriate N applications are essential for poplars to improve plant growth and N utilization efficiency (Franklin et al., 2016). In this study, two poplar clones XQH (*Populus psedosimonii* × *P. nigra* cv. Baicheng- 1) and BC5 (*Populus* × *Xiaozhanica* cv. Baicheng-5), which are widely afforested in Northern China and have high tolerance to infertile soil, were selected as the materials. It was hypothesized that (1) fertilization patterns (a total amount of N supply and conventional and exponential N additions) were the primary factors determining plant N status and utilization efficiency of different poplar trees; (2) leaf levels of PAs and photorespiration were implicated in poplar response to soil N availability. Special attention was focused on storage N (free amino acids, soluble proteins, PAs, and photorespiratory N) allocations in leaves of the poplar clones in the growing season of post- (conventional and exponential) fertilization year.

## Materials and Methods

### Plant Material

Two poplar clones (XQH and BC5) were propagated from 2-year-old cuttings. Cuttings of each clone were cultivated in 8.0-L pots filled with 2:1:1:1 (v:v:v:v) black soil–vermiculite–perlite–silver sand (total N: 0.86 g.kg^−1^, total P: 0.42 g.kg^−1^, available P: 32.15 mg.kg^−1^, organic matter: 22.76 g.kg^−1^) at Tree Breeding Garden of Northeast Forestry University, Harbin city, China. The clones were watered with tap water (about 600 ml per pot) every two days. Physical and chemical properties of tap water were described in detail in the Water Quality Report (Harbin Water Supply Group Co., Ltd 2018).

### Experimental Design

Two experiments were set up to assess (1) growth performance and leaf N utilization under five N-supply levels and (2) photosynthetic C and N metabolisms and excitation energy allocations in leaves under conventional and exponential N additions.

#### Experiment 1 (Exp. 1)

After plant growth for 2.5 months, seedlings of each poplar clone were separated into five groups (12 seedlings per group): the control (CK) group: seedlings grown without N addition during the growing season; the N2, N3, N4, and N5 groups: seedlings grown with 2, 3, 4, and 5 g N (in the form of urea, CH_4_N_2_O) addition per plant. N addition was conducted on July 8, July 28, and August 18 in 2017, respectively. On the first fertilization, P (1.430 g pot^−1^; Ca(H_2_PO_4_)_2_.H_2_O) and K (0.133 g pot^−1^; K_2_SO_4_) fertilizers were applied. Leaf N concentration, photosynthetic, and growth parameters were measured from July 8, 2017 at an interval of 20 days.

#### Experiment 2 (Exp. 2)

Based on the results of Exp. 1 and our previous experimental results (Ahmed, 2019), a total amount (6 g pot^−1^) of N fertilizer was used as an optimal N amount in Exp. 2. 1.5 months old seedlings of each poplar clone were separated into three groups (60 seedlings per group): the control (CK) group: seedlings without N addition during the growing season, (CF) group: seedlings with a conventional N addition (by two times), and (EF) group: seedlings with an exponential N addition (by six times). On the first fertilization (June 20, 2018), N (3 g N pot^−1^ for CF, and 0.387 g pot^−1^ for EF; the N form of CH_4_N_2_O), P (1.430 g pot^−1^; Ca(H_2_PO_4_)_2_.H_2_O), and K (0.133 g pot^−1^; K_2_SO_4_) fertilizers were applied. Each amount of exponential N addition was calculated on the formula *N*_t_ = *N*_s_ × (e*^rt^* − 1) − *N*_(t − 1)_ (Hawkins et al., 2005), where *N*_t_ is the amount of N to be added at time t for a given relative addition rate *r*, *N*_s_ the initial N content in plant, and *N*_(t − 1)_ is the cumulative amount of N added up to and including the last fertilizer addition (Ingestad and Lund, 1986). Exponential N fertilization was conducted at an interval of 10 days (from June 20 to August 10, 2018), and conventional fertilization was conducted on June 20 and July 20 in 2018, respectively. Before sampling, non-destructive measurements [shoot height (SH), basal diameter (BD), leaf area (LA), leaf photosynthesis) were conducted. For the measurements of organ metabolite concentration, key enzyme activity, and fresh organ (leaves, stem barks, and roots), samples were separately collected in growing seasons of the fertilizing year (on July 10, July 30, 2018) and post-fertilization year (May 24 and June 20, 2019), respectively. Samples collected were stored immediately in fresh boxes with ice packs. After washing with distilled water and surface-drying, all samples were stored at −80°C for future use.

#### Growth Traits

In Exp. 1 and Exp. 2, SH and BD were measured using a ruler and a vernier caliper, respectively. Leaf area (5^th^ to 7^th^ leaves from the top) of each plant was calculated by a grid count method. Fresh weight (FW) of plant organs was measured; dry weight (DW) was measured after oven-drying at 90°C to a constant mass. Absolute water content (AWC) of shoots was calculated as described by Ghashghaie et al. (1992): AWC = (FW − DW)/DW.

#### Gas Exchange Rates and Chlorophyll Index

In Exp. 1 and Exp. 2, photosynthetic parameters of the 5^th^–7^th^ fully-expanded leaves (from the top) were measured by using a LI-6400 portable photosynthesis system (LI-COR Bioscience, Inc., NE, USA). Net CO_2_ assimilation rate (Pn), stomatal conductance (*g*_s_), and transpiration rate (Tr) were determined at 380 μl.L^−1^ CO_2_, 65% RH, and 1,400 μmol.m^−2^.s^−1^ PPFD (photosynthetic photon flux density). Measurements were performed in the mornings between 9:00 a.m. and 11:00 a.m. Chlorophyll index (SPAD value) of the midpoint of the 3^rd^–5^th^ fully-expanded leaves (from the top) was measured by a SPAD 502 Plus Chlorophyll Meter (Minolta Camera Co., Osaka, Japan).

#### Leaf N Concentration and Utilization Efficiency

In Exp. 1, about 100-mg dried samples were treated by H_2_SO_4_–H_2_O_2_ Kjeldahl digestion method. Total N was determined by an indophenol method; the detailed procedures were described by Ohyama et al. (1991). Photosynthetic N utilization efficiency (PNUE) and transpiration-water N utilization efficiency (WNUE) were calculated by the following equations:

$$\begin{array}{l} \text{PNUE}=\text{Pn}/(\text{N}\times \text{DW/LA})\\ \text{WNUE}=\text{Tr}/(\text{N}\times \text{DW/LA}) \end{array}$$

Where DW is leaf dry weight (g), LA is leaf area (m^2^), N is leaf N concentration (%), Pn is net photosynthetic rate (μmol.m^−2^.s^−1^), Tr is transpiration rate (mmol.m^−2^.s^−1^).

#### Chlorophyll Fluorescence Variables

In Exp. 2, in the growing season of post-fertilization year (2019), chlorophyll fluorescence parameters of poplar leaves were measured using a portable fluorometer (M-PEA, Hansatech, King’s Lynn, UK). Leaves were first dark-adapted for 30 min, then exposed to a saturating red light pulse (650 nm, 3,000 μmol photons.m^-2^.s^-1^) provided by an array of six light-emitting diodes. Chlorophyll fluorescence variables were calculated automatically in Handy PEA v 1.3 software (Hu et al., 2019). CS: excited cross-section, RC: the reaction centers (RCs) of photosystems; ABS/CSo: absorption flux per CS at t = 0, which expresses antenna chlorophyll per CS, ABS/RC: absorption flux per active RC, DIo/CSo: dissipated energy flux of PSII per CS at t = 0, DIo/RC: dissipated energy flux per RC, ETo/ABS: quantum yield of electron transport between the two photosystems, ETo/CSo: electron transport flux per CS at t = 0, ETo/RC: electron transport flux per active RC, Fo/Fv: the efficiency of the oxygen-evolving complex (OEC) of PSII, RC/CSm: density of active RCs of PSII per excited CS.

#### Free Amino Acids, Soluble Sugars, and Soluble Proteins

In Exp. 2, fresh samples (about 100 mg power) were hydrolyzed in deionized water (1 ml); homogenates were transferred to a 1.5-ml centrifuge tube and boiled at 95°C for 15 min, then cooled with tap water. The tubes were then centrifuged at 8,000 × *g* at 4°C (total free AAs) or 25°C (soluble sugars) for 10 min. For soluble proteins, homogenate was centrifuged at 10,000 × *g* at 4°C for 10 min. Solution concentration was determined separately using free AAs, soluble sugars, and soluble protein assay Kits (Comin Biotechnology Co. Ltd, Suzhou, China) according to the manufacturer’s instructions. Metabolite concentration was calculated according to linear regression equations of the standard curve.

#### Composition Analysis of Free Amino Acids

In Exp. 2, fresh samples (about 150 mg power) were hydrolyzed in 1 ml deionized water. Samples were diluted and derivatized as done by Gao et al. (2017). The combined supernatant was labeled with iTRAQ reagents (API 20AA kit) and quantified by HPLC–MS/MS (UltiMate 3000 [Thermo Fisher Scientific Inc., Waltham, MA, USA]–API 3200 QTRAP [AB Sciex, Boston MA, USA]) using MSLAB HP-C18 column (150 mm long, 4.6 mm diameter, 5 μm particle size; Beijing Amino Acid Medical Research Co.). The detailed information on parameters and operations was described by Hu and Chen (2020).

#### Tricarboxylic Acids

In Exp. 2, fresh samples (about 150 mg power) were hydrolyzed in 1 ml deionized water. Then 50 μl solution was added to 200 μl of methanol (containing the internal standards). After standing for 1 min, samples were centrifuged at 13,000 × *g* at 4°C for 4 min. The supernatant was consequently collected and analyzed by HPLC-MS/MS (Ultimate3000-API 3200 Q TRAP) with ESI in negative ion mode. Chromatographic separation was conducted on a MSLab HP-C18 column (150 °C 4.6 mm, 5 μm; a flow rate of 1 ml·min^−1^; column temperature of 50°C). Two solvents, solvent A (water with 2 mmol·L^−1^ ammonium formate) and solvent B (acetonitrile with 2 mmol·L^−1^ ammonium formate), were used as mobile phases. Elution was operated as described by Gao et al. (2017). Mass spectrometry conditions were based on the descriptions by Jin et al. (2016).

#### Free Polyamine Analysis

In Exp. 2, fresh samples (about 150 mg power) were hydrolyzed in 1.5-ml 5% (w/v) perchloric acid. Samples were centrifuged at 15000 × *g* at 4°C for 30 min, and the supernatant was kept for further analysis. Free polyamines (putrescine: Put, spermidine: Spd, and spermine: Spm) and acetylated polyamines (A-Put, A-Spd, and A-Spm) were determined by HPLC-MS/MS (Ultimate3000-API 3200 Q TRAP) using a MSLab HP-C18 column (150 ×4.6 mm, 5 μm). The detailed measurements were described by Hu et al. (2019).

#### Activity of Glycolate Oxidase and Catalase, andH_2_O_2_ Concentration

In Exp. 2, the activity of key enzymes (CAT: catalase and GO: glycolate oxidase) was separately assayed by using CAT and GO assay kits (Comin Biotechnology Co. Ltd, Suzhou, China). The detailed measurements were operated according to the manufacturer’s instructions. Enzyme activity was calculated based on the absorbance values and a standard curve.

Fresh samples (100 mg leaf powder) were mixed with 1 ml acetone in ice bath. The homogenates were centrifuged at 8 000 × *g* at 4°C for 10 min. The supernatant was determined by monitoring the absorbance of the titanium–peroxide complex (binding between T_i_^4+^ and H_2_O_2_) at 415 nm. The detailed measurements were operated according to Zhao and Shi (2009).

### Statistical Analyses

Normality of data distribution and homogeneity of variance were first checked using the Shapiro–Wilk test and Levene’s test, respectively. Logarithmic transformation was applied if data did not meet the assumptions of normality and homogeneity of variance. Two-way analysis of variance (ANOVA) followed by Duncan’s test was used to examine the differences between experimental treatments. Differences were considered significant at *P* < 0.05. Non-parametric test (Kruskal–Wallis test) was applied for data of logarithmic transformation that still did not meet normality distribution and homogeneity of variance. Correlations between the excitation energy fractions (such as ABS/CS and DIo/CS) and polyamine or photorespiratory metabolic parameters were estimated using Pearson’s correlation coefficient (*r*) at the significant level of 0.05. The analyses were performed using Excel software (Microsoft Office Standard 2013, Microsoft, Redmond, WA, USA) and IBM SPSS Statistics 20.0 (IBM Corp., Armonk, NY, USA).

## Results

### Leaf N Concentration and Utilization Efficiency in Both Clones Under Different N Supply Levels

**Figure 1:**
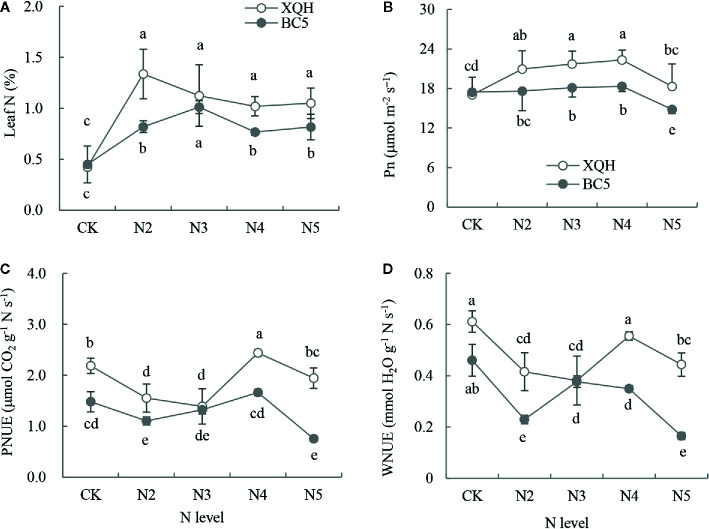
Leaf N concentration (%, **A**), net photosynthetic rate (Pn, **B**), photosynthetic N utilization efficiency (PNUE, **C**) and transpiration-water N utilization efficiency (WNUE, **D**) of two poplar clones (XQH and BC5) under different N-supply levels. CK: seedlings grown without N addition; N2, N3, N4, and N5: seedlings grown with 2, 3, 4, and 5 g N (in form of urea) per plant, respectively. An accumulative amount of N addition is 0 (CK), 4 g (N2), 6 g (N3), 8 g (N4), and 10 g (N5) per plant, respectively. The same lowercase letter above the error bars indicates no significant difference (*p* < 0.05; Duncan’s test).

N concentration significantly increased in fertilized compared to unfertilized seedlings of both XQH and BC5 clones (**Figure 1A**). Leaf N was significantly higher in seedlings of the BC5 clone under N3 than under the other N-supply levels; in contrast, leaf N was not different for the XHQ clone between N-supply levels. N concentration was not statistically different between the two clones under the unfertilized condition (CK) but was higher in the XQH than in the BC5 clone under N2, N4, and N5 conditions.

Pn was significantly higher for both clones under N3 and N4 compared to that under CK and under N5 (**Figure 1B**). Pn value was not different between the two clones under CK and was significantly higher in the XQH clone than in the BC5 clone under N3, N4, and N5. PNUE and WNUE significantly decreased in seedlings of both XQH and BC5 clones under N2 and N3 compared to that under CK. PNUE and WNUE were higher for both polar clones under N4 than under the other N-supply levels (**Figures 1C, D** **)**. PNUE and WNUE were higher in seedlings of XQH than of the BC5 clone under the same N-supply level (except for N3).

### Total Leaf Area, Shoot Dry Weight and Absolute Water Content in Both Clones Under Different N Supply Levels

**Figure 2:**
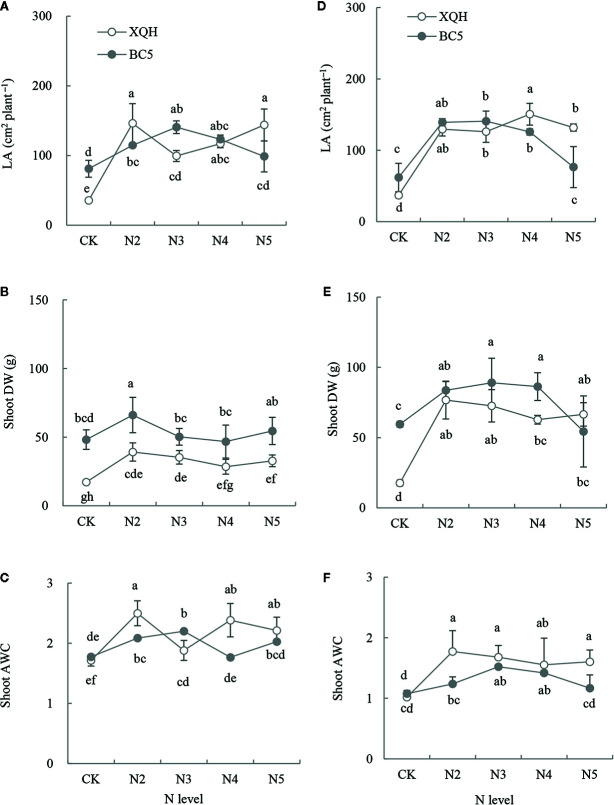
Leaf area (LA; **A**, **D**), shoot dry weight (DW; **B**, **E**) and absolute water content (AWC; **C**, **F**) of two poplar clones (XQH and BC5) under different N-supply levels. CK: without N addition; N2, N3, N4, and N5: with 2, 3, 4, and 5 g N (in form of urea) per plant, respectively. **(A–C)** represented the accumulative amount of N addition of 0 (CK), 4 g (N2), 6 g (N3), 8 g (N4), and 10 g (N5) per plant; **(D–F)** represented the accumulative amount of N addition of 0 (CK), 6 g (N2), 9 g (N3), 12 g (N4), and 15 g (N5) per plant. The same lowercase letter above the error bars indicates no significant difference (*p* < 0.05; Duncan’s test).

Total LA significantly increased in seedlings of both XQH and BC5 clones under the fertilized (except BC5 clone under N5) compared to unfertilized conditions (**Figures 2A, D**). LA was higher for the XQH clone under N2 (**Figure 2A**) or N4 (**Figure 2D**) and for the BC5 clone under N3 compared to the other N supply levels. LA was significantly higher in seedlings of the XQH than of the BC5 clone under N4 and N5 (**Figure 2D**).

Shoot DW was higher in both clones under fertilized (except BC5 clone under N5) than unfertilized conditions (**Figures 2B, E** **)**. Shoot DW was significantly lower in the XQH than in the BC5 clone under CK. Shoot DW was significantly different between XQH and BC5 clones under the same N-supply level in the second fertilization (**Figure 2B**) but not different in the third fertilization (**Figure 2E**). Shoot AWC increased in both poplar clones under the fertilized compared to unfertilized conditions (**Figures 2C, F**). The AWC values decreased in the third (**Figure 2F**) compared to the second fertilization (**Figure 2C**). Shoot AWC was significantly higher in the BC5 clone under N3 than under CK and N5 conditions. Shoot AWC was significantly higher in the XQH than in the BC5 clone under N2.

### Growth Performance of Two Poplar Clones Under Conventional and Exponential N Applications

Growth performances (SH, BD, and LA) were better for the XQH and BC5 clones under fertilized than unfertilized conditions in the growing season of fertilizing (2018) and post-fertilization (2019) years. There were no differences in the growth parameters of the XQH and BC5 clones between CF and EF treatments. SH was significantly higher in the XQH than in the BC5 clone with the same N supply in both fertilizing and post-fertilization years (**Table 1**).

**Table 1:** Growth and physiological parameters of two poplar clones (XQH and BC5) under conventional (CF) and exponential (EF) N applications in growing seasons of the fertilizing (2018) and post-fertilization year (2019).

Poplar	Fertilization	Jul 30, 2018						Jun 20, 2019						
		SH	BD	LA	Pn	AAs	SPs	SH	BD	Pn	SSs	OAs	AAs	SPs
	CK	112.11 ± 8.30^b*^	8.98 ± 0.91^b*^	46.58 ± 2.78^c^	13.59 ± 1.37^b^	9.81 ± 1.57^e^	90.20 ± 5.99^a^	164.00 ± 4.08^c*^	14.76 ± 0.79^b*^	15.18 ± 1.15^a^	59.24 ± 5.43b	929 ± 110^bc^	35.87 ± 0.44^bc^	142.16 ± 7.95^c^
XQH	CF	145.55 ± 12.16^a*^	12.24 ± 1.38^a*^	110.38 ± 9.36^a^	19.43 ± 2.32^a^	41.37 ± 5.33^a^	53.67 ± 3.40^b^	242.33 ± 18.32^a*^	20.65 ± 1.19^a*^	13.65 ± 2.41^a^	73.92 ± 2.71^a^	1232 ± 58^ab^	40.07 ± 5.04^ab^	121.13 ± 8.48^d^
	EF	145.40 ± 20.03^a*^	11.83 ± 1.85^a*^	106.34 ± 17.26^a^	20.33 ± 1.91^a^	34.83 ± 3.28^b^	51.28 ± 3.83^c^	239.33 ± 21.12^a*^	20.65 ± 1.83^a*^	13.55 ± 1.38^a^	70.39 ± 4.24^a^	1390 ± 66^a^	44.38 ± 3.33^a^	111.66 ± 6.42^d^
	CK	88.57 ± 7.12^d*^	8.62 ± 0.83^b*^	42.24 ± 4.72^c^	16.14 ± 0.66^b^	16.05 ± 2.66^d^	95.90 ± 6.69^a^	127.30 ± 9.55^d*^	12.84 ± 1.01^b*^	10.75 ± 2.06^b^	56.42 ± 3.48^b^	916 ± 292^bc^	32.80 ± 2.20^c^	180.31 ± 14.24^a^
BC5	CF	102.32 ± 7.80^b*^	10.32 ± 1.04^a*^	97.66 ± 8.80^ab^	20.36 ± 2.26^a^	21.10 ± 2.78^d^	72.06 ± 5.76^b^	216.60 ± 6.54^b*^	21.45 ± 2.64^a*^	17.11 ± 1.37^a^	67.72 ± 2.90^a^	868 ± 234^bc^	37.10 ± 1.92^bc^	167.85 ± 10.04^ab^
	EF	101.30 ± 7.77^b*^	11.18 ± 0.64^a*^	89.46 ± 8.73^b^	19.90 ± 0.95^a^	26.87 ± 3.79^c^	88.82 ± 6.10^a^	209.00 ± 6.87^b*^	20.53 ± 1.63^a*^	15.90 ± 1.33^a^	71.24 ± 2.43^a^	815 ± 152^c^	40.22 ± 4.27^ab^	162.26 ± 11.38^b^

CK, seedlings without N application; SH, shoot height (cm); BD, basal diameter (mm); LA, leaf area (cm^2^); Pn, net photosynthetic rate (µmol·m^−2^·s^−1^); AAs, free amino acids μmol×g^-1^ FW); SPs, soluble proteins (mg·g^−1^ FW); SS, soluble sugars (mg·g^−1^ FW); OAs, organic acids in TCA cycle (µg·g^−1^ FW). Means with the same lowercase letter are not significantly different (p < 0.05; Duncan’s test). *indicates a significance analysis by using the non-parametric Kruskal–Wallis test (p < 0.05).

(mean ± SD; n = 10 for SH and LA, n = 4 for the other index). * indicates a significance analysis by using the non-parametric Kruskal-Wallis test (p < 0.05).

Leaf Pn significantly increased in fertilized compared to unfertilized seedlings of the BC5 clones in the fertilizing (2018) and post-fertilization (2019) years (**Table 1**). In contrast, leaf Pn was higher in fertilized than in unfertilized seedlings of XQH clone only in the fertilizing year (2018). Leaf Pn was not different for the two poplar clones between CF and EF treatments in the years 2018 and 2019.

### Leaf Soluble C and N Metabolites of Two Poplar Clones Under Conventional and Exponential N Applications

Free AAs concentration was higher, but SPs were lower in fertilized than unfertilized seedlings of the XQH clone in the years 2018 and 2019. Concentration of free AAs or SPs was significantly different between CF- and EF-treated XQH clones only in 2018. For the BC5 clone, free AAs concentration significantly increased only in EF-treated seedlings compared to CK in 2018 and 2019. SP concentration was significantly lower in CF-treated seedlings (in 2018) and EF-treated seedlings (in 2019) than in CK.

SS concentration was significantly higher in fertilized than unfertilized seedlings of the XQH and BC5 clones, without difference between the CF and EF seedlings. OA concentration was not different for the BC5 clone between treatments. OA concentration was significantly higher for the XQH clone under EF than CK condition.

### Leaf Polyamines Content in Two Poplar Clones Under Conventional and Exponential N Applications

Put concentration was significantly higher in the EF seedlings than in the control ones (for both XQH and BC5 clones) and CF seedlings (only for XQH clone) (**Table 2**). Leaf Put was significantly higher in the XQH than in the BC5 clone under both CK and EF conditions. Concentrations of Spd and Spm were not different for the XQH clone between treatments; in contrast, Spm concentration was significantly lower in the fertilized than in the unfertilized BC5 clones, without difference between CF and EF treatment.

**Table 2:** Leaf free polyamines concentration (μg·g^−1^ FW) of two poplar clones (XQH and BC5) supplied with conventional (CF) and exponential (EF) N in the growing season of post-fertilization year.

Poplar	Treatment	Polyamines			Acetylated polyamines		
		Put	Spd	Spm	A-Put	A-Spd	A-Spm
	CK	0.53 ± 0.21^b^	0.15 ± 0.02^b^	0.60 ± 0.05^c^	0.05 ± 0.02^b^	0.16 ± 0.11^a^	0.27 ± 0.13^a^
XQH	CF	0.69 ± 0.10^b^	0.17 ± 0.04^b^	0.61 ± 0.11^c^	0.09 ± 0.04^b^	0.17 ± 0.10^a^	0.30 ± 0.26^a^
	EF	1.79 ± 0.91^a^	0.16 ± 0.03^b^	0.72 ± 0.07^bc^	0.13 ± 0.08^a^	0.31 ± 0.20^a^	0.29 ± 0.06^a^
	CK	0.18 ± 0.08^c^	0.24 ± 0.06^a^	1.23 ± 0.44^a^	0.07 ± 0.05^b^	0.18 ± 0.11^a^	0.12 ± 0.07^b^
BC5	CF	0.44 ± 0.25^bc^	0.19 ± 0.04^ab^	0.89 ± 0.14^b^	0.08 ± 0.02^b^	0.22 ± 0.10^a^	0.13 ± 0.07^b^
	EF	0.48 ± 0.20^b^	0.22 ± 0.06^ab^	0.89 ± 0.14^b^	0.11 ± 0.04^ab^	0.19 ± 0.13^a^	0.15 ± 0.06^b^

CK, seedlings without N application; Put, putrescine; Spd, spermidine; Spm, spermine; A, acetylated. Means with the same lowercase letter are not significantly different (p < 0.05; Duncan’s test). (mean ± SD; n = 4).

Acetylated Put concentration significantly increased in the XQH clone under EF compared to CK and CF conditions; in contrast, acetylated Spd and Spm were not different between treatments. Acetylated polyamines were not different for the BC5 clone between treatments.

### Chlorophyll Fluorescence Parameters of Two Poplar Clones Under Conventional and Exponential N Applications

Leaf Chl index of the XQH clone significantly increased under the fertilized compared to unfertilized conditions, without differences between CF and EF treatments (**Table 3**). In contrast, leaf Chl was not different for the BC5 clone between treatments.

**Table 3:** Leaf chlorophyll fluorescence parameters of two poplar clones (XQH and BC5) in the first post-fertilization year.

Poplar	Fertilization	Chl (SPAD)	Cross-section based index			PSII reaction center based index			RC/CSm	ETo/ABS	Fo/Fv
			ABS/CSo	ETo/CSo	DIo/CSo	ABS/RC	ETo/RC	DIo/RC			
	CK	30.58 ± 2.47^b^	5355 ± 324^c^	1475 ± 538^c^	1189 ± 204^b^	1.23 ± 0.15^a^	0.33 ± 0.08^a^	0.28 ± 0.07^ab^	20446 ± 5210^b^	0.27 ± 0.10^b^	0.29 ± 0.05^a^
XQH	CF	40.37 ± 4.11^a^	6251 ± 273^b^	2375 ± 254^a^	1284 ± 138^b^	1.06 ± 0.09^ab^	0.40 ± 0.03^a^	0.22 ± 0.04^b^	29316 ± 4463^a^	0.38 ± 0.04^a^	0.26 ± 0.03^a^
	EF	38.87 ± 4.82^a^	6752 ± 372^a^	2122 ± 322^ab^	1634 ± 203^a^	1.16 ± 0.12^ab^	0.36 ± 0.03^a^	0.28 ± 0.06^ab^	24788 ± 5634^ab^	0.31 ± 0.04^ab^	0.32 ± 0.05^a^
	CK	34.78 ± 2.26^ab^	5781 ± 319^bc^	1901 ± 326^b^	1370 ± 70^b^	1.27 ± 0.31^a^	0.41 ± 0.07^a^	0.30 ± 0.09^a^	20204 ± 4709^b^	0.33 ± 0.04^ab^	0.31 ± 0.04^a^
BC5	CF	30.92 ± 3.39^b^	5760 ± 195^bc^	2088 ± 272^ab^	1222 ± 112^b^	0.99 ± 0.05^b^	0.36 ± 0.04^a^	0.21 ± 0.02^b^	27516 ± 3087^a^	0.36 ± 0.05^a^	0.27 ± 0.02^a^
	EF	35.90 ± 4.21^ab^	6059 ± 249^b^	2112 ± 265^ab^	1362 ± 196^b^	1.10 ± 0.13^ab^	0.38 ± 0.05^a^	0.25 ± 0.06^ab^	25139 ± 4957^ab^	0.35 ± 0.05^a^	0.29 ± 0.05^a^

Chl, chlorophyll; CS, excited cross-section; RC, the reaction centers (RCs) of photosystems; ABS/CSo, absorption flux per CS at t = 0, which expresses antenna chlorophyll per CS; ABS/RC, absorption flux per active RC; DIo/CSo, dissipated energy flux of PSII per CS at t = 0; DIo/RC, dissipated energy flux per RC; ETo/ABS, quantum yield of electron transport between the two photosystems; ETo/CSo, electron transport flux per CS at t = 0; ETo/RC, electron transport flux per active RC; Fo/Fv, the efficiency of the oxygen-evolving complex (OEC) of PSII; RC/CSm, density of active RCs of PSII per excited CS. Means with the same lowercase letter are not significantly different (p < 0.05; Duncan’s test). (mean ± SD; n = 6).

For the XQH clone, ABS/CSo and DIo/CSo significantly increased under EF compared to CK and CF conditions. ETo/CSo was significantly higher in the fertilized than in the unfertilized seedlings. In contrast, the above chlorophyll fluorescence parameters were not different for the BC5 clone between treatments. The PSII reaction center-based indexes (ABS/RC, ETo/RC, and DIo/RC) were not different for the XQH clone between treatments; whereas the values of ABS/RC and DIo/RC were significantly lower for the BC5 clone only under CF (than CK) condition. RC/CSm was significantly higher for both XQH and BC5 clones only under CF (than CK) condition. ETo/ABS and Fo/Fv were not different for both poplar clones between treatments.

### Leaf Photorespiration of Two Poplar Clones Under Conventional and Exponential N Applications

Activity of GO significantly increased in leaves of XQH clone under the fertilized compared to the unfertilized controls, with significantly higher values under EF than CF conditions (**Table 4**). In contrast, GO activity was not different in the leaves of fertilized and unfertilized seedlings of BC5 clone.

**Table 4:** Leaf photorespiratory levels of two poplar clones (XQH and BC5) supplied with conventional and exponential N application in the first post-fertilization year.

Poplar	Fertilization	GO	CAT	H_2_O_2_ (μmol·g^−1^ FW)	Gly (μg·g^−1^ FW)	Gly : Ser	Glu : Gln	MA (μg·g^−1^ FW)
		(nmol·min^−1^·mg^−1^ prot)						
	CK	0.46 ± 0.02^d^	0.47 ± 0.05^bc^	95.38 ± 5.46^b^	4.24 ± 0.24^a^	0.38 ± 0.04^bc^	34.90 ± 16.03^ab^	484.81 ± 18.04^b^
XQH	CF	0.82 ± 0.10^c^	0.33 ± 0.03^d^	80.31 ± 5.12^d^	5.28 ± 1.44^a^	0.43 ± 0.05^b^	18.50 ± 6.58^b^	1014.43 ± 69.45^a^
	EF	1.25 ± 0.22^a^	0.42 ± 0.05^c^	76.71 ± 7.74^d^	5.98 ± 1.56^a^	0.55 ± 0.09^a^	43.02 ± 0.26^a^	1103.63 ± 52.47^a^
	CK	0.89 ± 0.09^bc^	0.85 ± 0.05^a^	126.15 ± 4.28^a^	2.70 ± 0.84^b^	0.25 ± 0.08^d^	28.73 ± 4.64^ab^	240.74 ± 66.99^c^
BC5	CF	0.82 ± 0.08^c^	0.31 ± 0.03^d^	125.10 ± 4.78^ab^	2.52 ± 0.41^b^	0.27 ± 0.07^cd^	33.74 ± 6.19^ab^	369.85 ± 98.38^b^
	EF	1.07 ± 0.16^ab^	0.52 ± 0.06^b^	116.68 ± 6.76^b^	2.69 ± 0.82^b^	0.27 ± 0.08^cd^	27.06 ± 14.75^b^	281.60 ± 33.17^b^

CAT, catalase; Gln, glutamine; Glu, glutamic acid; Gly, glycine; GO, glycolate oxidase; H_2_O_2_, hydrogen peroxide; MA, malic acid; Ser, serine. Means with the same lowercase letter are not significantly different (p < 0.05; Duncan’s test). (mean ± SD; n = 4).

H_2_O_2_ concentration was significantly lower in the XQH and BC5 clones under EF compared to CK conditions, without difference between CF and EF treatments. CAT activity significantly decreased in both clones under CF compared to CK and EF conditions with a significant difference for the BC5 clone between EF and CK conditions.

Glycine concentration was not different for the two clones between treatments, with significantly higher values in the leaves of the XQH than BC5 clone. Glycine-to-serine ratio significantly increased in the XQH clone under EF compared to CF and CK conditions. Glutamate-to-glutamine was lower in the CF- than EF-treated XQH clone, without differences between fertilized and unfertilized seedlings for both poplar clones. MA concentration significantly increased in the fertilized compared to the unfertilized seedlings of both clones.

## Discussion

Organic N fertilizers (such as urea) are commonly used in the management of poplar seedlings. Organic N application significantly improved poplar growth (Coleman et al., 2006; Zhang et al., 2018). In our study, an interaction of the clone and N supply was found significant in the growth performance of poplar plants. Clone XQH performed better than BC5 under N supply by exhibiting enhanced growth attributes and N utilization. BC5 clone is more sensitive to N than XQH clone (**Figures 1** and **2**). Leaf morphology and physiology are significantly important in plant adaptation to alternations of the environmental factors (including soil N availability). Under N supply, increasing N allocation to leaves is an adaptive strategy for plants to improve photosynthetic C and N use efficiency (Perchlik and Tegeder, 2018). However, in our study, PNUE and WNUE significantly decreased but N concentration significantly increased in the leaves of both XQH and BC5 clones under low N supply compared to the unfertilized control. This result implies that the N allocated to the leaves may be used preferentially for leaf expansion, which is indicated by a significant increase of the leaf area. The storage N such as nitrate and water-soluble proteins are the regulatory hub of leaf expansion and photosynthetic capacity (Liu et al., 2018). During leaf expansion, the storage N in the leaves are primarily used for cell division and chloroplast development (Roggatz et al., 1999). The N allocation strategy is applicable to our results that low N supply (N2 or N3) increased N allocation to the leaves, further stimulating leaf expansion. However, growth inhibition occurred in the BC5 clone at high N supply (N5); this may be due to the restrictions of root growth and N uptake as well as subsequent N transport upward to shoots (White, 2012).

Soluble proteins and amino acids are considered major N sources for leaf expansion. In general, leaf concentration of SPs decreases along with leaf expansion (Liu et al., 2018). Our results showed that leaf area significantly increased in the fertilized than unfertilized seedlings of both XQH and BC5 clones, without difference in leaf area between CF and EF (**Table 1**). Leaf AAs and SPs are significantly different in the XQH clone under both EF and CK conditions but not different under CF and CK conditions in post-fertilizing year. There were no statistical differences in the storage N in the seedlings of the BC5 clone under conventional and exponential N additions in the post-fertilizing year. Our results confirmed a higher continuity of storage N utilized by poplar trees under exponential compared to conventional N addition. Leaf level of the storage N is an effective index in evaluating the real efficacy of different N application (such as CF and EF) models. Compared to the storage N, the growth parameters (such SH, BD, and shoot biomass) are ineffectual in distinguishing the difference in poplar responses to N-application patterns (Zabek and Prescott, 2007; and the present study).

Polyamines are significantly important in the regulation of cell division, protein synthesis, and other cellular processes in leaves (Nieves et al., 1998; Alcázar et al., 2010). The availability of N influences the cellular PA biosynthesis in plants. For example, cellular PAs (putrescine, spermidine, and spermine) significantly decreased in plants (rice) under N deficiency compared to N supply (Sung et al., 1995). Moreover, cellular PAs in the plants (such as tobacco and poplar) were significantly affected by supplemental N forms (*e.g.*, the ratio of nitrate to ammonium nutrition, Altman and Levin, 2006; the ratio of nitrite to nitrate, Hu et al., 2019). Our results indicate that exponential additions of the organic N (urea) significantly increased leaf Put concentration of the XQH and BC5 poplar clones but decreased leaf Spm of the BC5 clone. Putrescine is a central metabolite of PA metabolism, closely linked with amino acids, organic acids, and hormonal cross-talks (Hu and Chen, 2020). In previous studies (*e.g.* Li et al., 2015), Put conversion to higher PAs (such as Spd and Spm) is considered as an adaptive strategy for the plants to environmental alterations. However, excess accumulation of higher PAs in the cells may be toxic, at least in part, to plasma membranes (Capell et al., 2004; Hu and Chen, 2020). Our results support the above view because light absorption index (ABS/CSo) has a negative correlation with Spm concentration (*r* = −0.80, *p* = 0.05; **Figure S1**), in contrast to a positive correlation with Put concentration (*r* = 0.74, *p* = 0.09). Polyamine acetylation possibly through the conjugation with hydroxycinnamic acids is another important mechanism in the regulation of cellular PA level (Luo et al., 2009). Our results confirmed the significant occurrence of PA acetylation (acetylated Put in the XQH poplar clone with exponential N addition; **Table 2**). We inferred that Put acetylation may be implicated in dissipation of excess excitation energy captured by light-harvesting complex II because of a significant positive correlation between acetylated Put and the dissipated energy flux (DIo/CSo; **Figure S1**). However, only the correlation analysis is not adequate to confirm the correlations between PAs and the photochemical processes. A systematic study (through using PA-associated mutants and/or the inhibitors or stimulators specially related to PA metabolism and photochemical processes) or the direct evidence is needed to confirm the close correlations and uncover the underlying mechanisms in poplar response to changes of soil N availability.

In this study, photorespiration is responsive to organic N additions, particularly for the XQH clone; leaf GO activity and Gly/Ser ratio significantly increased under exponential compared to conventional N additions. Photorespiration is significantly important in the regulation of leaf C and N metabolisms through the glyoxylate and glycine recycling (Wang et al., 2014), Glu-involved metabolic pathways (*i.e.*, the biosynthesis of proteins and amino acids; Hu et al., 2019), and mitochondrial malate dehydrogenase-mediated tricarboxylic acid cycle (Tomaz et al., 2010). Photorespiratory N cycle [ammonia release through glycine decarboxylation and reassimilation by chloroplastic glutamine synthetase (GS2)] is significantly faster than primary nitrogen assimilation (Keys et al., 1978). For the GS2-deficient mutants, the inability to reassimilate photorespiratory ammonium resulted in leaf accumulation of free ammonium (*e.g.*, tobacco; Oliveira et al., 2002), even the whole plant death (*e.g.*, barley; Blackwell et al., 1987). The study by Oliveira et al. (2002) has demonstrated a close correlation between the levels of cytosolic GS1 and photorespiration and inferred that the efficiency of reassimilation of photorespiratory ammonium was an important factor determining NUE and plant growth (Oliveira et al., 2002). Our study confirmed significant alternations of Gly-to-Ser ratio and Glu-to-Gln ratio in poplar leaves when plants were subjected to exponential (compared to conventional) N addition. However, a direct link of photorespiration with NUE or plant growth is unavailable in our results. A recent study using NO_3_^−^ as N source reported the photorespiration-mediated stimulation on leaf CO_2_ assimilation (Busch et al., 2018); in contrast, under NO_3_^−^ deficiency, both the photosynthetic and photorespiratory rates decreased. In our study, leaf photosynthetic and photorespiratory levels are significantly higher in the fertilized than the unfertilized poplar clones. Several studies have demonstrated the importance of photorespiration in regulating leaf allocation of excitation energy in plants (such as *Schima superba, Crytocarya concinna*, and *Populus alba × Populus berolinensis*) with N addition (*e.g.*, Cai et al., 2008; Hu et al., 2014). Our results showed significantly positive correlations of photorespiration (GO activity) with light absorption (ABS/CSo) and thermal dissipation (DIo/CSo) processes (**Figure S2**). However, metabolomics and transcriptome experiments are needed to uncover the real roles of photorespiration in complex regulation networks in plant response to N addition.

## Conclusions

Poplar growth is significantly reduced under the limitation of soil N availability. Supplemental N application significantly stimulated leaf expansion, photosynthetic C and N metabolisms, and shoot growth. The efficacy of N supply largely depends on an interaction of the clone and N application (a total N amount and a conventional or exponential N model). The storage N (such as AAs and SPs) may be significantly important in the regulation of leaf expansion in poplar adaptation to exponential N addition. Allocation of excitation energy in photosystem II, the polyamines (Put and the acetylated Put) and photorespiratory metabolism (GO activity, Gly-to-Ser ratio, and Glu-to-Gln ratio) are implicated in poplar response to soil N availability.

## Data Availability Statement

The raw data supporting the conclusions of this article will be made available by the authors, without undue reservation.

## Author Contributions

YH and XZ designed the experiment, analyzed the data, and wrote the paper. MS revised the manuscript. YH, CL, LJ, and HZ conducted the experiment. All authors contributed to the article and approved the submitted version.

## Funding

The study was financially supported by National Key Research and Development Program of China (Grant No. 2016YFD0600404); Heilongjiang Province Postdoctoral Research Starting Fund (LBH-Q19002); the Open Fund of Key Laboratory of Dryland Agriculture, Ministry of Agriculture, P. R. China (2018KLDA02).

## Conflict of Interest

The authors declare that the research was conducted in the absence of any commercial or financial relationships that could be construed as a potential conflict of interest.
