# Supplementary material for: Polyamine Metabolism, Photorespiration, and Excitation Energy Allocation in Photosystem II Are Potentially Regulatory Hubs in Poplar Adaptation to Soil Nitrogen Availability
Source: Front Plant Sci. 2020 Aug 26;11:1271. doi: 10.3389/fpls.2020.01271 (PMC7479266; doi:10.3389/fpls.2020.01271)
Supplement: Supplementary file 1 [file Table_1.docx]

**Fig. S1** Correlations between polyamines and chlorophyll fluorescence parameters in leaves of the poplar clones (XQH and BS5) under N additions. A-Put: acetylated putrescine, Put: putrescine, Spm: spermine; ABS/CSo: absorption flux per CS at t=0, Chl: chlorophyll, DIo/CSo: dissipated energy flux of PSII per CS at t=0. The correlation was analyzed by using Pearson correlation coefficient (*r*, at a significance level of 0.05)
